# Factors Affecting Weigh Tape Reading in the Measurement of Equine Body Weight

**DOI:** 10.3390/ani13081330

**Published:** 2023-04-13

**Authors:** Katie Grimwood, Bryony Lancaster, Ian Handel

**Affiliations:** 1 Baileys Horse Feeds, Four Elms Mills, Braintree, Essex CM7 5EJ, UK; 2 The Royal (Dick) School of Veterinary Studies, The University of Edinburgh, Midlothian EH25 9RG, UK; bryony.lancaster@ed.ac.uk (B.L.); ian.handel@ed.ac.uk (I.H.); 3 The Roslin Institute, The University of Edinburgh, Midlothian EH25 9RG, UK

**Keywords:** weigh tape, bodyweight, estimation, horse, body condition scoring, equine

## Abstract

**Simple Summary:**

Different methods can be used for measuring a horse’s body weight, including a specific equine weigh tape, which gives a body weight estimate based on the heart girth circumference. Since more accurate equine weighbridges are not easily available due to high cost, weigh tapes are a common method of estimating body weight in horses. However, this measurement is not always accurate, with different factors potentially affecting tape readings. Therefore, the aim of this study was to investigate which variables affect weigh tape readings. Using a large data set, available through a commercial horse feed company, the results suggested that weigh tape readings may be influenced by breed, bone density, and body fat coverage. These results will help give a greater understanding and aid in the interpretation of weigh tape measurements in the future.

**Abstract:**

Accurate measurement of equine body weight is important for evaluating medication dosages and feed quantities. Different methods exist for measuring body weight, including weigh tapes (WT), though accuracy varies. Measurements could be affected by external variables, such as time of day, human error, or uneven surfaces, and also horse-based variables, such as height and body condition score (BCS). The aim of this study was to investigate how different horse-based variables affect WT reading. A retrospective analysis was performed using anonymised data from feed company nutrition consultations (Baileys Horse Feeds). Data included a range of horse-based variables, a WT reading, and true body weight measured on a weighbridge. All horses were over two years of age. Likelihood ratio tests were used to assess whether adding different horse-based variables significantly improved the fit of the quadratic regression model. The variables included were height, BCS, breed, muscle top-line score, and bone type. Exploratory analysis showed that the WT generally underestimated body weight, particularly for horses with higher body weight. Adding height and muscle top-line scores did not significantly improve the fit of the model, suggesting no influence on WT reading over and above actual body weight. Adding breed groupings, BCS, and bone density did improve the fit. Each 0.5 unit increase in BCS increased the WT estimate by 1.24 kg (*p* < 0.001). These results confirm that a WT does not provide accurate body weight measurements, and generally underestimates body weight, though more so for heavier horses, being more accurate in pony breeds.

## 1. Introduction

An accurate measurement of a horse’s body weight is important for medication and anthelmintic dosages, as well as quantities of feed and forage provided. An incorrect estimation may therefore be detrimental to the horse’s health. Underdosing of anthelmintics may result in the survival of resistant parasites, increasing the chances of this genetic trait flourishing and parasitic damage [1]. An incorrect medication dosage may also have serious implications, depending on the medication used. In a large retrospective study, 67.2% of horses had received an inadequate antimicrobial dosage prior to colic surgery, which was suggested to have been due to incorrect bodyweight measurements, resulting in incorrect dosage calculations [2].

Under or overfeeding may cause weight loss or weight gain. Excess weight gain may increase the chances of developing issues such as laminitis [3] and can increase loading pressure on the limbs, potentially contributing to the development of osteoarthritis [4]. Additionally, both excess weight loss and weight gain can negatively affect performance [5,6]. An unsuitable feed provision may also lead to excesses or deficiencies in nutrients, the balance of which is fundamental to the body’s health and function [7]. For example, deficiencies in iron, copper, or cobalt may result in reduced haemoglobin, leading to a decreased oxygen-carrying capacity [8].

An equine weighbridge is the most accurate method to measure body weight, but these are not always easily available, due to the high cost [9]. Visual estimation has been shown to be consistently inaccurate, with one study finding that owners misjudged their draft and warmblood horses’ body weight by an average of 52 kg [10]. Therefore, it is not recommended as a suitable assessment of body weight [11]. In goats, body structure and coat colour have been noted to have a subconscious effect on visual estimations of body weight [12], and this may also apply when assessing the horse. Weight estimation formulae, based on body length and heart girth [13], can be used and this may be a more accurate method [11,14]. However, this method may have practical limitations in busy equine establishments, as it can be more time-consuming. The addition of shoes must also be considered for height measurements, and two people are required to measure body length.

Therefore, specific equine weigh tapes may be used as an alternative. These give an estimated bodyweight reading based on heart girth circumference, with a reading taken following exhalation, and can be easily performed by one person. Although the weigh tape offers a more accessible and affordable method to horse owners and requires only one measurement, its very nature means that the accuracy is reduced, as variations in individual body shapes will not be considered [14,15]. Measurements could be affected by external variables, like time of day, human error, or an uneven surface, but could also be influenced by horse-based variables which are harder to control. It has previously been shown that breed can affect the accuracy of different weight estimation formulae in horses [16] and that incorporating breed, height, and other morphometric measurements into a weight estimation formula increases the accuracy of body weight prediction [17]. It is therefore plausible that these factors may also influence weigh tape reading. Another variable which also requires further investigation is the influence of body condition, or fat coverage, on the accuracy of the weigh tape. Wagner and Tyler [15] identified that body condition score (using the 1–9 method from Henneke et al. [18]) tended to be a predictor of body weight, but it is not yet clear as to whether this may influence weigh tape reading in bodyweight estimations.

The objective of this study was to investigate which different horse-based variables affect weigh tape reading using a single, commercially available weigh tape.

## 2. Materials and Methods

A retrospective analysis was carried out using existing data collected from eight feed company nutrition consultants (Baileys Horse Feeds, Essex, UK) over two years and eight months, from January 2017 to August 2019. The study was ethically approved by the Human (Research) Ethical Review Committee (reference was HERC 247-18), R(D)SVS, University of Edinburgh. Owners of the horses gave consent for the data to be recorded and stored by Baileys Horse Feeds, who gave permission for the data to be used in a fully anonymised data set to the researcher for the study. As retrospective data was used, the selection of horses was not standardised and was dependent on operational limitations as well as the nature of the nutrition consultation (such as whether the owners wanted their horses weighed).

**Figure 1:**
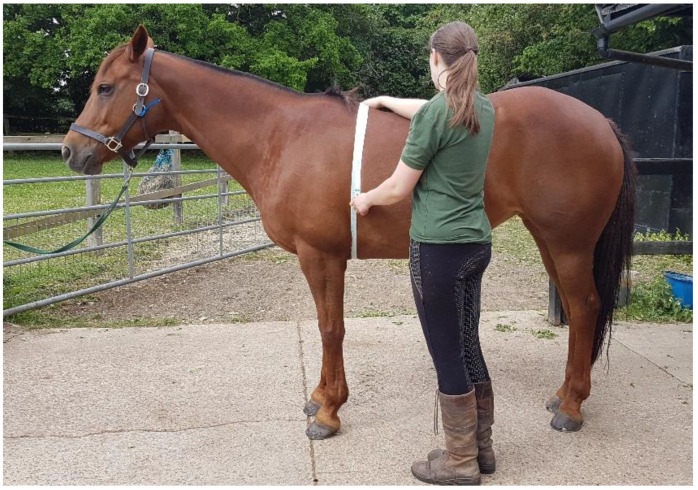
The correct placement of the weigh tape around the circumference of the horse’s girth.

Details on the horse’s height and breed were obtained from the owner or handler, and the assessors assigned a muscle top-line score using a standardised muscle top-line assessment scoring system developed by the company [19]. This system gives a score based on the individual’s overall muscle development and top line over the neck, withers, back, hip, and stifle. Scores range from ‘poor’, whereby the horse’s musculature is extremely weak, through to ‘excellent’, which describes ideal muscle development and definition over the spine with no areas of weakness. A body condition score was also assigned, using the 1–9 system devised by Henneke et al. [18]. The assessors also observed (based on prior experience) whether the horse would be classed as a ‘heavy weight’, ‘medium weight’, or ‘light weight’ bone type, though this was not standardised. Data also included a weigh tape reading, using the same type of weigh tape in all cases (Shires Equestrian Horse & Pony Weighband). The tape is made of polyvinyl chloride (PVC) and has a maximum measuring length of 206 cm. To take the measurement, the weigh tape was placed behind the point of the elbow and in a straight vertical line over the withers, around the circumference of the horse’s girth, with the reading taken following exhalation (Figure 1). All measurements were carried out on a level surface with the horse standing square and holding a relaxed head carriage. A true bodyweight was also recorded, and this was measured on a calibrated weighbridge (Newmarket Wireless Portable 3 Piece Horse Weigher). Although different weighbridges were used by different consultants, they were all the same brand and model and were calibrated using the same procedure. The weighbridges had a maximum load capacity of 2000 kg and were correct to the nearest 1 kg. All measurements for each individual horse were taken within the same period during each nutrition consultation. All consultants were fully trained and experienced in the procedures involved, including the use of the weighbridge and the weigh tape.

The data set provided by the feed company contained 2675 records. However, 959 records contained incomplete or missing information which was required for analysis, such as breed, weigh tape weight, or weighbridge weight. This may have been because the breed was not known by the owner or handler, or if the horse did not tolerate either the weigh tape or walking onto the weighbridge. These records were therefore removed prior to analysis, leaving a total of 1716 records for analysis. All the horses were over the age of two years old. Whilst records were also available for youngstock measurements (under the age of two years), these were not included as part of the analysis, due to the relatively small number of records available.

Exploratory plots were initially created to get an understanding of the data and the relationship between weigh tape readings and actual weights. The relationship was evaluated using both linear and quadratic regression models. The quadratic models were included, as it was possible the weigh tape might not have a ‘straight line’ relationship with the actual weight. The models were then compared using a likelihood ratio test, which compares how well the models fit whilst adjusting for their complexity.

Other variables were then considered to determine if these had any effect on the relationship between weigh tape and true body weight. Variables included were height, body condition score, breed, muscle top-line score, and bone type. Bland Altman plots were used to show the relationship of the weigh tape and actual weight, compared to the actual weight, for the different variables, as this method of plotting allows for easier identification of trends as well as outliers [20]. These variables were then incorporated into the regression model to investigate whether they improved the goodness of fit. For hypothesis tests comparing models and assessing the significance of estimates, a critical *p* value of 0.05 was used.

## 3. Results

### 3.1. Summary Statistics

One hundred and sixty different breeds or cross-breeds were represented (Figure S1, Table S1), though for analysis purposes, these were divided into four different categories, based on those previously described [14]: small ponies under 350 kg (*n* = 237, 13.8%) (SP); large ponies more than 350 kg but under 14.2 hh/147 cm (*n* = 322, 18.8%) (LP); lighter builds of horses, such as thoroughbreds (*n* = 701, 40.9%) (LH); and stockier breeds of horses such as drafts (*n* = 456, 26.6%) (SH). Horses in the LH and SH groups were all 14.2 hh/147 cm and above. In total, 32.6% were ponies (under 14.2 hh/147 cm) and 67.4% were horses (14.2 hh/147 cm and above). This is consistent with previously identified demographics of the UK horse population [21] and so it may be considered a representative sample.

Table 1 shows the summary statistics for the sample population and within each breed category. The overall mean weigh tape reading was 32.82 kg lower than the overall mean actual weight (range −151–64 kg). Compared to true bodyweight, the weigh tape had an average error of −5.68% (95% CI [−6.01%, −5.34%]).

### 3.2. Exploratory Plots and Regression Modelling

**Figure 2:**
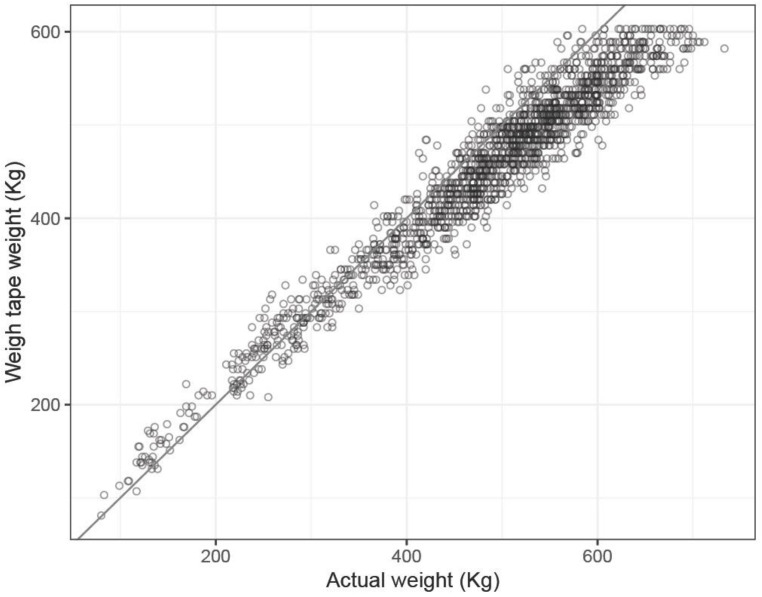
The weigh tape readings compared to the actual weights of 1716 horses. The grey line is an ‘identity line’, where those points which perfectly agree would lie.

Initially, a scatterplot was created to get a basic understanding of how the weigh tape compared to the actual weight (Figure 2). This indicated that there is a strong relationship between the weigh tape and the actual weight, but that the weigh tape generally underestimates the weight, particularly for heavier horses.

To better understand the relationship between the weigh tape reading and the actual weight, a straight-line regression model was initially fitted to the data. However, since the data shown above in Figure 2 has a slight curve, a second, quadratic, regression model was also tried, which incorporated the square of the actual weight to give a curved line model.

The two models were compared using a likelihood ratio test to assess the goodness of fit of both models. A likelihood ratio test assesses if adding a variable (or variables) significantly improves the fit of the model. The quadratic ‘curved line’ model had a significantly better fit to the data than the simple linear regression model (*p* < 0.001). This also suggests that the relationship between the two variables is non-linear, in that a change in one does not correspond with a constant change in the other.

### 3.3. Height

Height was then added to the model to see if this would improve the fit. However, there was no evidence that height added anything extra to the prediction over and above the actual weight (*p* = 0.417).

### 3.4. Body Condition Score

**Figure 3:**
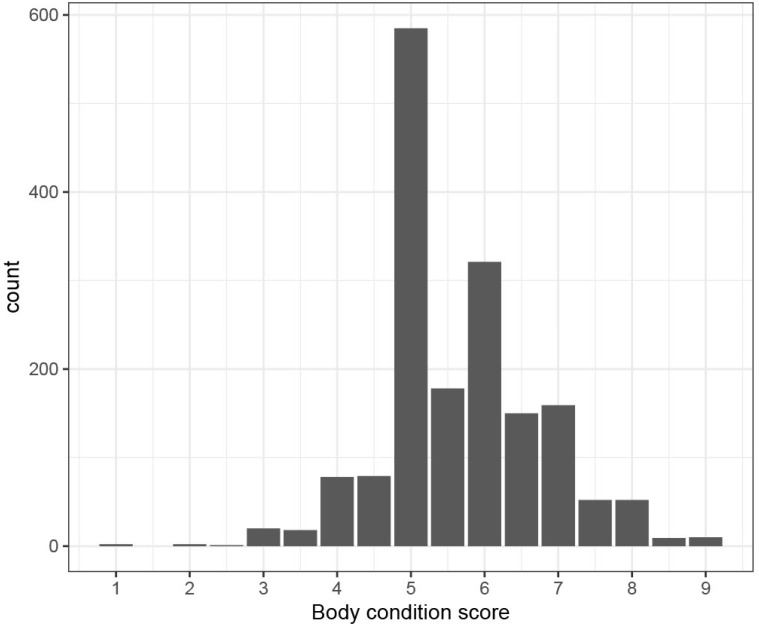
The distribution of body condition scores of 1716 horses using the 1–9 scale [18].

**Figure 4:**
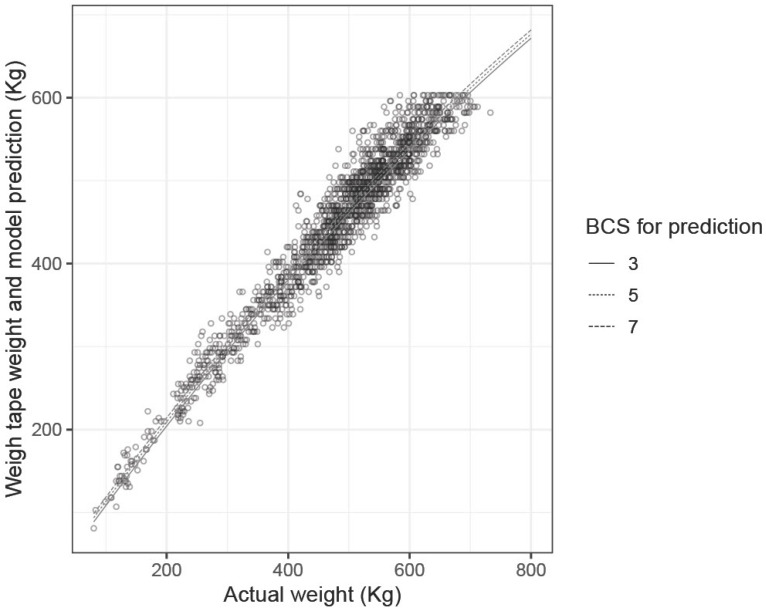
The improved prediction of horse bodyweight using the quadratic regression model with the inclusion of body condition score [18] within the model.

Body condition scores were normally distributed but were skewed towards a higher score (Figure 3), though the majority of horses had a score of between four and six out of nine (72%). Table 2 shows the average error of the weigh tape for each body condition score unit. When the body condition score was included within the regression model (Figure 4), this was found to improve the prediction. The model estimated that for every 0.5 increase in body condition score unit, the weigh tape on average will add an extra 1.24 kg to the reading (in horses with the same actual body weight) (*p* < 0.001).

### 3.5. Breed

**Figure 5:**
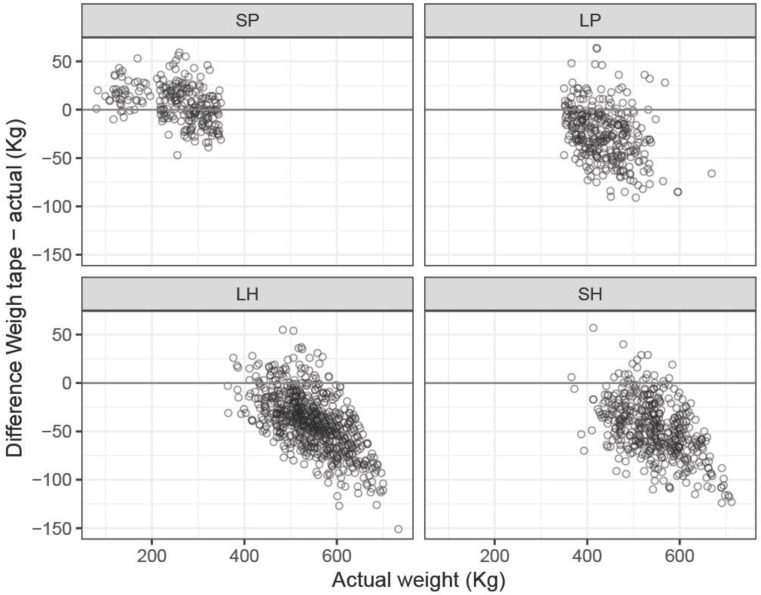
The relationship of the difference between the weigh tape and actual weights, compared to the actual weights, separated into four breed categories [14], which were: small ponies under 350 kg (SP); large ponies more than 350 kg but under 14.2 hh/147 cm (LP); lighter builds of horses such as thoroughbreds (LH); and stockier breeds of horses such as drafts (SH).

Bland Altman plots were created for the four breed categories (Figure 5). These showed that for the horses in the SH category, the weigh tape tended to underestimate, whereas, for the horses in the SP category, the weigh tape tended to overestimate slightly, and was also more centred around the horizontal line which represented no difference. This is also supported by the variations in weigh tape error shown in Table 1. Adding breed groupings improved the fit of the quadratic model (*p* < 0.001), with up to 1.87 kg differences on average between breed groups.

### 3.6. Muscle Top Line Score

**Figure 6:**
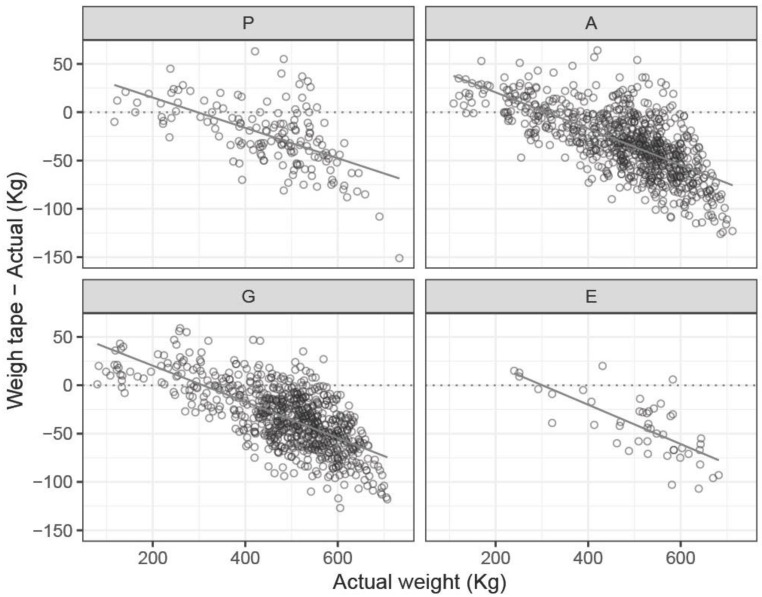
The relationship of the difference between the weigh tape and actual weights, compared to the actual weights, separated into muscle top line score. P = ‘poor’, A = ‘adequate’, G = ‘good’, E = ‘excellent’ [19].

The muscle top-line scores were also assessed using Bland Altman plots (Figure 6). This indicated that for the ‘excellent’ top line score, the weigh tape slightly underestimated body weight compared with the other scores. However, when this was added to the regression model, the prediction was not improved (*p* = 0.442).

### 3.7. Bone Type

**Figure 7:**
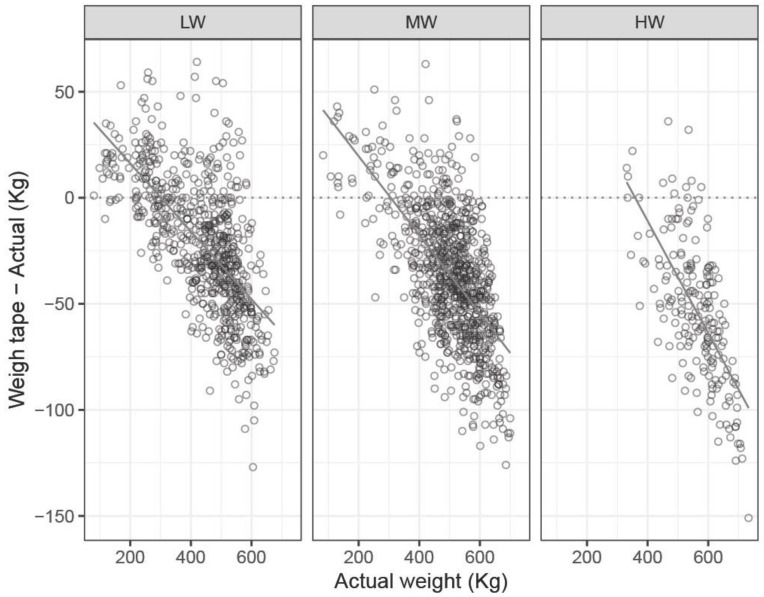
The relationship of the difference between the weigh tape and actual weights, compared to the actual weights, separated into bone type. LW = light weight bone, MW = medium weight bone, HW = heavy weight bone.

Bone type was also considered as part of the regression model and this did improve the fit of the prediction of body weight (likelihood ratio test, *p* = 0.00039). Heavy weight and medium weight boned horses were found to have a lower weigh tape error compared to light weight boned horses (Table 3, Figure 7).

## 4. Discussion

This study aimed to explore possible factors influencing weigh tape readings in a large group of horses, using a single, commercially available weigh tape. The results supported the hypothesis that there would be a difference between weigh tape readings and true body weight. Deviations from true bodyweight ranged from a difference of 1–2 kg, through to differences of over 100 kg. Whilst the smaller deviations are less relevant in practice, with these slight under- or overestimations being less likely to affect the horse’s well-being, the larger deviations may be more problematic in practice. Overall, the weigh tape was found to have an average error of −5.68%, which corresponds with previous results [22,23]. Ellis and Hollands [22] recorded weigh tape readings ranging from an underestimation of 3% to an overestimation of 12% for horses under 14.2 hh, and from an underestimation of 9.2% to an overestimation of 2.6% for horses over 14.2 hh, when comparing three different tapes (including one height-specific tape). Different weigh tape brands have minor differences in their formulae for estimating body weight based on girth circumference, though this background information is held by the manufacturer and is not accessible in the public domain [15]. The three tapes used by Ellis and Hollands [22] were different brands to the Shires Equestrian Horse & Pony Weighband used in the present study, but the evidence together suggests that the accuracies of other brands of tape may be likely to also fall within this error range.

Wagner and Tyler [15] used yet another different brand of weigh tape and found that, although the weigh tape still underestimated, there was a difference between mean actual weights and mean weigh tape weights of 65.81 kg. In the present study, the mean difference was 32.82 kg, which was less than half that found by Wagner and Tyler [15]. However, differences in the study population will likely have affected the results. For example, Wagner and Tyler [15] specifically excluded pony breeds, whilst the present study included a much wider range of breeds and sizes. Since the results of the present study suggested that breed can influence weigh tape reading and that the weigh tape may be more accurate for smaller pony breeds, it is perhaps unsurprising that the overall mean kilogram difference between weigh tape and actual weight was much smaller. However, since the breed categories in the present study also corresponded with the weight of the horse, the results of the present study may be just due to bodyweight alone rather than breed specifically (as shown in Figure 2 with heavier horse weights tending to be underestimated on the weigh tape). Future research could look at comparing weigh tape readings in different breeds of horses that all had a similar true body weight.

The findings that larger, heavier horses tended to be underestimated on the tape and smaller, lighter horses were more accurate may be due to the fact that longer or stockier legs and other body proportions are not taken into account, as the tape measures girth circumference only. Previous research has found that the accuracy of a single weigh tape was lower in horses over or equal to 15 hh, at 91.8%, but improved to 99% accuracy when considering those under 15 hh [11]. In the present study, heavy and medium weight boned horses had lower weigh tape readings than light weight boned horses, although the method of bone weight assessment was not standardised. However, these results are supported by another study which found that ‘stockier’ horse weights were underestimated by 15%, whereas pony weigh tape readings were relatively accurate [14]. Future studies could use a more standardised method of determining bone type, such as measuring cannon bone circumference, which was unfortunately not possible in the present study, given its retrospective nature.

The results of the present study also indicated body condition scores can influence weigh tape readings. This expands on previous work by Wagner and Tyler [15], who identified that body condition score was a key potential factor in predicting body weight, but no specific research has previously been carried out to determine its influence on weigh tape readings. These results indicate that the weigh tape formula may favour a larger girth circumference relative to the size and proportions of the rest of the body and that knowledge of a horse’s body condition score using the 1–9 method [18] may be a useful factor in gauging the accuracy of the weigh tape in field conditions. Considering the results of the present study, since the weigh tape generally underestimates, it is possible that overweight horses (those with a body condition score of six and above) are perhaps more likely to have a more accurate weigh tape reading. However, since the majority of horses in the study were within the body condition score range of four and six, with fewer individuals being extremely underweight or extremely overweight, further research using horses with a more even spread of body condition scores may be required to validate this hypothesis. Despite the variations in accuracy and the influence of other factors, weigh tapes may still prove a useful and accessible tool in bodyweight estimations and particularly in supporting owner motivation to continue managing weight loss in overweight equines [24].

As this study was retrospective, there were some limitations. Considering the large data set, not all weigh tape measurements were performed by the same individual, and records from eight different assessors were used. This means that there may have been some slight observer bias, with minor differences between assessors in how they took the measurements. It was not possible to test for the effect of different assessors as it was not noted in the data which measurements were recorded by which individual. The assessors were also not required to perform weigh tape measurements prior to using the weighbridge, so were effectively not blinded to the horses’ actual weights. This may have resulted in a degree of unconscious subjectivity when positioning the weigh tape and taking the reading. However, previous research has argued that changes in positioning and tension of the weigh tape contribute only a minor role in the error in weigh tape estimation, as they will only affect the measurement by just a few centimetres [14]. Furthermore, since all assessors were fully trained and experienced in the use of the weigh tape, and all used the same brand of weigh tape, this is less likely to have significantly affected the results.

The other limitation of using an existing data set is that not all factors were able to be investigated, including both environmental factors and additional horse-based factors. All the horses were over the age of two, due to insufficient records available for younger horses, and the specific ages of those included were not known, other than that they were all over two years. Whilst this does follow the protocol of previous research where those aged under two years were also excluded [15], it may be seen as a limitation as well, as age may also have a potential impact on weigh tape accuracy, due to conformational changes as the horse matures [25]. Gender was also not recorded within the data set, so, again, this could not be investigated. In a study by Souza et al. [26], it was found that a commercial weigh tape in Brazil was more accurate in male Campolina horses than in females. A previous study in 2017 using the same brand of weigh tape found that it overestimated female horses by an average of 7.4 kg and underestimated the weights of male horses by an average of 7.5 kg [27]. However, 40% of the mares studied were pregnant at the time of measuring, and stage of pregnancy can also affect weigh tape readings [28]. Furthermore, of the non-pregnant subjects (male and female) in the 2017 study, 68% were under the age of five years (minimum of six months), so age-related conformational changes may not have been considered [25]. It has been suggested that a young age may influence bodyweight predictions based on heart girth in both horses and other animals, and that weigh tapes are typically designed for use in mature equines [29,30]. Therefore, more research may still be required into the influence of gender and age on weigh tape reading, and these would be factors to incorporate if this study was to be repeated. Other factors for a future observational study could include body length and neck circumference, as these have been noted to influence weight estimation formulae [17]. In time, it may be possible to develop different weigh tapes with different formulae for specific breeds or types, and this may be useful for improving accuracy.

## 5. Conclusions

These results demonstrate that a weigh tape cannot be relied upon to provide an accurate measurement of body weight. The weigh tape generally underestimates body weight, though more so for horses with a higher true body weight. In horses with a lower body weight, the weigh tape may provide a more accurate reading. Horse-based variables, which improved the fit of the quadratic regression model and therefore may affect weigh tape reading, include body condition score, breed, and bone density. Horses with a higher body condition score may be more likely to have a more accurate weigh tape reading compared to horses with a lower body condition score. Muscle development and height did not improve the fit of the regression model over and above actual body weight, and, therefore, these factors do not appear to affect weigh tape reading. Further research could aim to consider additional horse-based variables, as well as accounting for variations in different brands of weigh tape.

## Figures and Tables

**Table 1:** Summary statistics for 1716 horse records overall and within each breed category [14].

	Overall ^1^	Small Ponies < 350 kg (SP)	Large Ponies ≥ 350 kg but <14.2 hh/147 cm (LP)	Lighter Horse Breeds (LH)	Stockier Horse Breeds (SH)
height (cm)	149.43 (18.58)	115 (18.21)	139.45 (4.99)	161.98 (7.95)	155.07 (7.2)
body condition score [18]	5.7 (1.07)	5.8 (1.22)	6 (1.04)	5.2 (0.84)	6 (1.07)
actual weight (kg)	484.37 (119.01)	254.56 (66.91)	435.99 (52.45)	544.84 (65.17)	545.01 (64.07)
weigh tape reading (kg)	451.55 (99.92)	261.08 (62.2)	410.84 (50.23)	504.53 (52.47)	497.84 (55.39)
average weigh tape error (%)	−5.68 (7.06)	3.83 (8.96)	−6 (6.05)	−7.11 (4.93)	−8.47 (5)

^1^ Mean (standard deviation).

**Table 2:** Average weigh tape error for each body condition score unit.

Body Condition Score [18]	Average Weigh Tape Error (%) ^1^
1	−3.64 (5.14)
2	1.98 (5.71)
3	−4.07 (6.87)
4	−6.07 (5.86)
5	−6.38 (6.63)
6	−5.32 (7.54)
7	−4.63 (7.06)
8	−2.24 (8.73)
9	5.11 (9.35)

^1^ Mean (standard deviation).

**Table 3:** Average weigh tape error for each bone type category.

Bone Type	Average Weigh Tape Error (%) ^1^
light weight	−3.62 (7.53)
medium weight	−6.52 (6.52)
heavy weight	−9.17 (5.27)

^1^ Mean (standard deviation).

## Data Availability

Restrictions apply to the availability of these data. Data was obtained from Baileys Horse Feeds and is available from the corresponding author with the permission of Baileys Horse Feeds.

## Supplementary Materials

The following supporting information can be downloaded at: https://www.mdpi.com/article/10.3390/ani13081330/s1, Figure S1: Chart to show the 10 most frequent horse breeds represented in the date. Table S1: Details of the breeds and cross-breeds represented in the data and the number of horses within each.
