# Supplementary material for: Factors Affecting Weigh Tape Reading in the Measurement of Equine Body Weight
Source: Animals (Basel). 2023 Apr 13;13(8):1330. doi: 10.3390/ani13081330 (PMC10135119; doi:10.3390/ani13081330)
Supplement: Supplementary file 1 [file animals-13-01330-s001.zip › animals-2314810-supplementary.pdf]

Supplementary Data

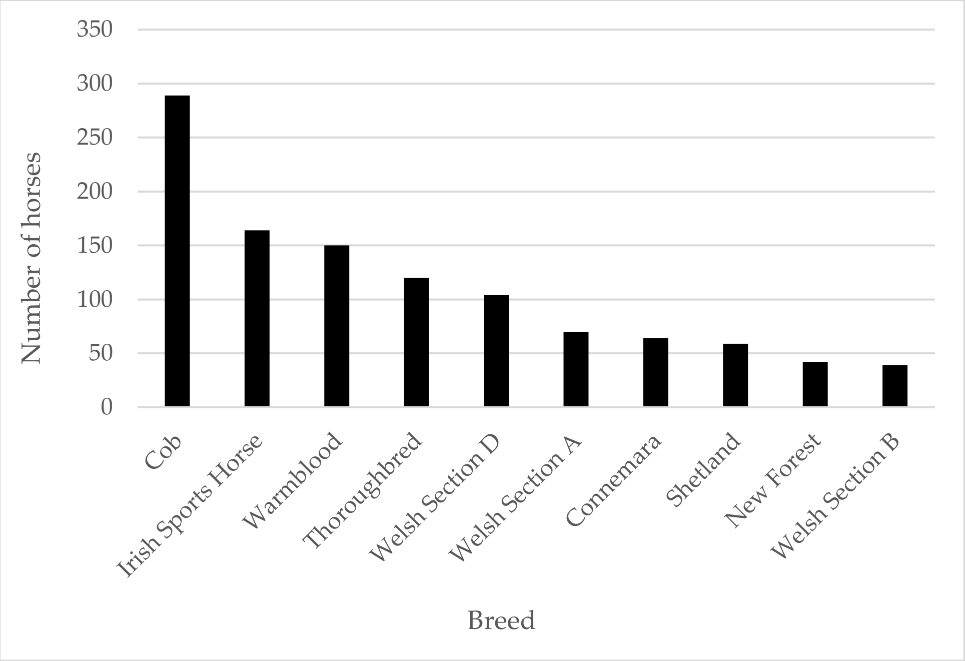

Supplementary Figure S1. Chart to show the 10 most frequent horse breeds represented in the data.

Supplementary Table S1. Details of the breeds and cross-breeds represented in the data and the number of horses within each.

Breed or cross-breeds	Number of horses
Andalusian	16
Andalusian x Arab	1
Andalusian x Cob	1
Andalusian x Unknown	3
Anglo Arab	4
Anglo European	4
Appaloosa	12
Arab	13
Arab x Appaloosa	1
Arab x Connemara	1
Arab x Dartmoor	2
Arab x Trotter	1
Arab x Unknown	7
Arab x Warmblood	2
Belgian Warmblood	11
British Riding Pony	20
Camargue x Unknown	1
Cleveland Bay x Thoroughbred	1
Cleveland Bay x Unknown	1

Clydesdale	2
Clydesdale x Connemara	1
Clydesdale x Thoroughbred	1
Clydesdale x Unknown	1
Cob	289
Cob x American Paint	2
Cob x Appaloosa	3
Cob x Arab	1
Cob x Clydesdale	1
Cob x Dales	1
Cob x Irish Draught	1
Cob x Standardbred	1
Cob x Welsh	2
Connemara	64
Connemara x Andalusian	1
Connemara x Cob	5
Connemara x Irish Sports Horse	7
Connemara x New Forest	1
Connemara x Saddlebred	1
Connemara x Thoroughbred	7
Connemara x Unknown	11
Connemara x Warmblood	3
Connemara x Welsh	3
Connemara x Welsh Section B	1
Dales	11
Dales x Cob	1
Dales x Irish Sports Horse	1
Dartmoor	17
Dartmoor x Shetland	1
Dartmoor x Unknown	1
Driving Pony (Unknown breed)	2
Exmoor	6
Fell	10
Fell x Dales	1
Fell x Unknown	2
Fell x Welsh Section D	1
Fjord	2
Friesian	4
Friesian x Cob	2
Friesian x Trotter	1
Friesian x Unknown	1
Hackney	1
Hackney x Thoroughbred	1
Hackney x Welsh Section D	1
Haflinger	14
Haflinger x Unknown	1

Hanoverian	8
Hanoverian x Cob	1
Hanoverian x Irish Sports Horse	2
Hanoverian x Thoroughbred	3
Hanoverian x Unknown	3
Hanoverian x Warmblood	1
Highland	6
Irish Draught	17
Irish Draught x Connemara	6
Irish Draught x Unknown	15
Irish Draught x Warmblood	5
Irish Sports Horse	164
Irish Sports Horse x Cob	7
Knabstrupper	3
Lipizzaner x Cob	2
Lipizzaner x Friesian	1
Lipizzaner x Unknown	1
Lusitano	5
Morgan x Cob	1
Morgan x Connemara	1
Native (Unknown)	10
New Forest	42
New Forest x Cob	2
New Forest x Thoroughbred	3
New Forest x Unknown	8
New Forest x Warmblood	1
Oldenburg	3
Oldenburg x Thoroughbred	1
Polo Pony	3
Pony (Unknown)	7
Quarter Horse	3
Riding Horse (Unknown)	5
Shetland	59
Shetland x Fell	1
Shetland x New Forest	1
Shetland x Unknown	1
Shire x Thoroughbred	1
Shire x Unknown	2
Show Pony x Cob	1
Sports Pony	9
Standardbred	7
Standardbred x Friesian	1
Thoroughbred	120
Thoroughbred x Appaloosa	2
Thoroughbred x Cob	16
Thoroughbred x Irish Sports Horse	4

Thoroughbred x Unknown	18
Thoroughbred x Warmblood x Clydesdale	1
Trakehner	3
Trakehner x Anglo Arab	1
Trakehner x Cob	1
Trakehner x Connemara	1
Trakehner x Unknown	1
Trotter	7
Trotter x Cob	9
Trotter x Unknown	2
Trotter x Welsh	3
Warmblood	150
Warmblood x Appaloosa	1
Warmblood x Cob	4
Warmblood x Irish Sports Horse	5
Warmblood x Sports Pony	1
Warmblood x Thoroughbred	20
Warmblood x Thoroughbred x Connemara	1
Warmblood x Thoroughbred x Welsh	1
Warmblood x Unknown	13
Welsh Section A	70
Welsh Section A x Cob	1
Welsh Section A x Irish Draught	1
Welsh Section A x Shetland	2
Welsh Section A x Unknown	1
Welsh Section B	39
Welsh Section B x Arab	3
Welsh Section B x Unknown	1
Welsh Section C	17
Welsh Section C x Cob	1
Welsh Section C x Unknown	1
Welsh Section D	104
Welsh Section D x Cob	2
Welsh Section D x Irish Draught	1
Welsh Section D x Lusitano	1
Welsh Section D x Thoroughbred	3
Welsh Section D x Thoroughbred x Irish Draught	1
Welsh Section D x Unknown	8
Welsh Section D x Warmblood	1
Welsh x Appaloosa	3
Welsh x Arab	10
Welsh x Dales	1
Welsh x Fell	1
Welsh x Hackney	1
Welsh x Irish Draught	1

Welsh x Thoroughbred	2
Welsh x Unknown	27
Welsh x Warmblood	1
Westphalian	9
